# Comparative genome analysis reveals a conserved family of actin-like proteins in apicomplexan parasites

**DOI:** 10.1186/1471-2164-6-179

**Published:** 2005-12-12

**Authors:** Jennifer L Gordon, L David Sibley

**Affiliations:** 1 Department of Molecular Microbiology, Washington University, School of Medicine, 660 S. Euclid Ave. St, Louis, MO, USA

## Abstract

**Background:**

The phylum Apicomplexa is an early-branching eukaryotic lineage that contains a number of important human and animal pathogens. Their complex life cycles and unique cytoskeletal features distinguish them from other model eukaryotes. Apicomplexans rely on actin-based motility for cell invasion, yet the regulation of this system remains largely unknown. Consequently, we focused our efforts on identifying actin-related proteins in the recently completed genomes of *Toxoplasma gondii, Plasmodium *spp., *Cryptosporidium *spp., and *Theileria *spp.

**Results:**

Comparative genomic and phylogenetic studies of apicomplexan genomes reveals that most contain only a single conventional actin and yet they each have 8–10 additional actin-related proteins. Among these are a highly conserved Arp1 protein (likely part of a conserved dynactin complex), and Arp4 and Arp6 homologues (subunits of the chromatin-remodeling machinery). In contrast, apicomplexans lack canonical Arp2 or Arp3 proteins, suggesting they lost the Arp2/3 actin polymerization complex on their evolutionary path towards intracellular parasitism. Seven of these actin-like proteins (ALPs) are novel to apicomplexans. They show no phylogenetic associations to the known Arp groups and likely serve functions specific to this important group of intracellular parasites.

**Conclusion:**

The large diversity of actin-like proteins in apicomplexans suggests that the actin protein family has diverged to fulfill various roles in the unique biology of intracellular parasites. Conserved Arps likely participate in vesicular transport and gene expression, while apicomplexan-specific ALPs may control unique biological traits such as actin-based gliding motility.

## Background

The phylum Apicomplexa contains several protozoan pathogens that cause severe disease in mammals, including humans. Members such as *Plasmodium falciparum*, and *P. vivax*, which cause severe human malaria, and *Theileria parva and T. annulata*, which are responsible for economic losses in cattle in Africa, result in profound medical, social, and economic effects [1,2]. Others such as *Toxoplasma gondii, Cryptosporidium parvum *and *C. hominis *are primarily health threats in HIV+/AIDS and immunosuppressed populations [3].

Apicomplexans are primarily obligate intracellular parasites that rely on actin-based motility for cell invasion [4]. Invasion occurs by active parasite motility that is coupled to timed secretion of proteins from specialized apical secretory organelles, which are a hallmark feature of this phylum [5,6]. The apical secretory organelles (called micronemes, rhoptries, and dense granules) release their contents in a highly regulated fashion upon host cell interactions [7]. Microneme proteins provide adhesion to the host cells and supply the traction needed for invasion. Rhoptry and dense granule proteins function in the establishment and maintenance of a protective, intracellular niche called the parasitophorous vacuole (reviewed in [8]). Understanding how motility and invasion are regulated is crucial to elucidating the pathobiology of these organisms, yet we know relatively little about how these functions are controlled at the cellular level.

Apicomplexans are characterized by a unique cytoskeleton that is distinct from that of other eukaryotes [9]. At their apical end is a specialized microtubule-organizing center called the polar ring complex, which coordinates a series of singlet microtubules called the subpellicular microtubules [10,11]. The remarkable stability of these microtubules provides a defined shape and polarity to the cells that is necessary for motility and invasion [12]. The subpellicular microtubules encompass the apical secretory organelles and may play a role in trafficking to the apical end of the cell. Apicomplexans also regulate their actin cytoskeleton differently, maintaining a large pool of soluble actin, both globular and in short, unstable filaments [13-15]. During motility, actin filaments must rapidly assemble to support gliding and then turnover rapidly to prevent unwanted movement. Actin regulation is thus crucial to the control of motility. In other eukaryotes, a large family of actin-related proteins helps control many cytoskeletal functions including vesicle transport and actin-based motility.

Actin-related proteins (Arps) are conserved across all eukaryotes and some prokaryotes. Although all members share a common actin-fold and an overall sequence similarity to actin [16-18], individual Arps carry out a variety of biochemical and structural roles in the cell [19]. These include roles in cell division [20], translocation of cargo along microtubules via dynein [21,22], actin polymerization [23], and transcriptional regulation via chromatin/ heterochromatin remodeling [24-26]. Currently, more than 11 classes of Arps have been reported from a broad range of eukaryotes including plants, animals, fungi, and protozoans (i.e. *Dictyostelium, Acanthamoeba*, and *Tetrahymena)*. In each case, the Arp groups link the separate kingdoms both by protein similarity and common biochemical functions. Despite their apparent conservation among the majority of eukaryotes, no Arps have been previously described in the Apicomplexa.

Complete genome sequences have recently been provided for a variety of apicomplexan parasites. A cursory examination of these genomes reveals multiple actins and actin related proteins; however, these have been inconsistently identified and annotated. The complex biology of these parasites led us to examine actin-related proteins in this phylum relative to other eukaryotes using a combination of phylogenetic and reciprocal BLAST analyses. Our findings reveal a complexity of actin-related proteins not previously appreciated and define both conserved and unique members of this protein family within the Apicomplexa.

## Results and discussion

### Phylogenetic comparisons of actin-like proteins in apicomplexans

**Figure 1:**
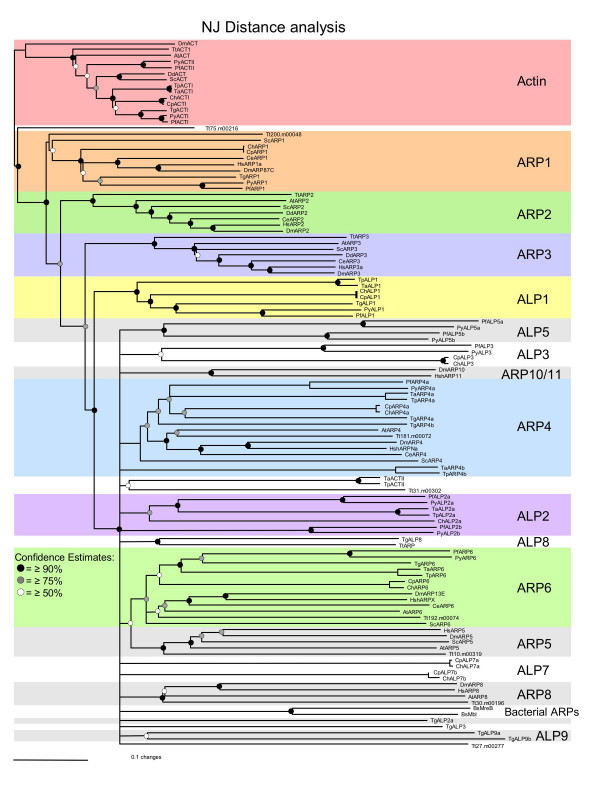
**Phylogenetic comparisons of actin and actin-related proteins in apicomplexans and model organisms. **In addition to conventional actins, apicomplexans contain conserved Arp1, Arp4, and Arp6 proteins; however, they do not encode Arp2 or Arp3 orthologues. Many apicomplexan proteins do not group with any of the known Arp clades. These have been divided further into proteins that are highly conserved among all the apicomplexans (i.e. ALP1, ALP2, ALP3) and those that are organism-specific (i.e. ALP5, ALP7, ALP8). Phylogenetic analysis was performed using PAUP*4.0b10 and the BioNeighbor-Joining algorithm (BioNJ) to determine the divergence distances among taxa. Consensus trees were bootstrapped for 1000 replicates and drawn according to the 50% majority-rule. Conventional actin was defined as the out-group. Subgroups of Arps and ALPs have been highlighted to define the boundaries between groups. Taxa are as follows: At = *Arabidopsis thaliana*, Bs = *Bacillus subtilis*, Ce = *Caenorhabditis elegans*, Cp = *Cryptosporidium parvum*, Dd = *Dictyostelium discodium*, Dm = *Drosophila melanogaster*, Hs = *Homo sapiens*, Pf = *Plasmodium falciparum*, Sc = *Saccharomyces cerevisiae*, Tg = *Toxoplasma gondii*, Tp = *Theileria parva*, Tt = *Tetrahymena thermophila*. Bootstrap values ≥90% are represented by the black nodes ●, values ≥ 75% are represented by the gray nodes , and values ≥50% are denoted by the white nodes ○.

**Figure 2:**
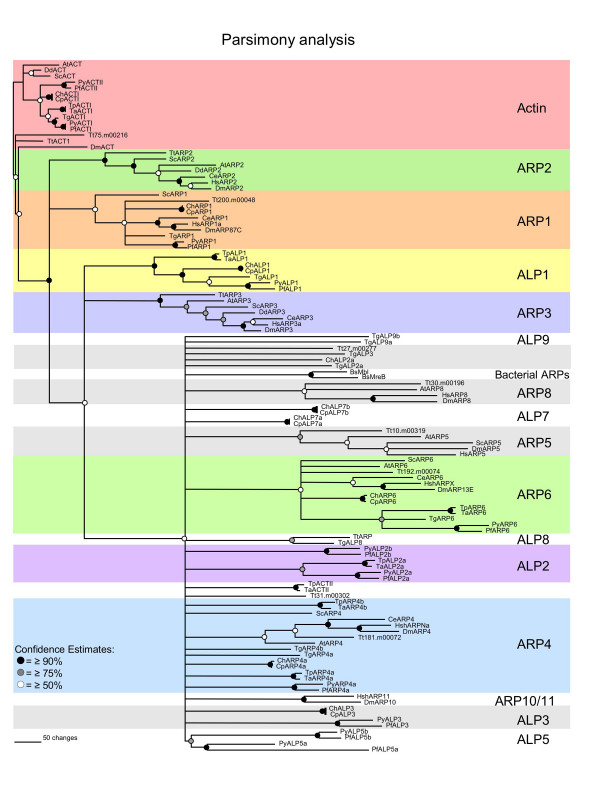
**Parsimony analysis of actin and actin-related proteins from apicomplexans and other taxa. **Phylogenetic analysis performed using parsimony resulted in groupings that mirrored distance analysis. All major classes of Arp and ALP groups are maintained except for the Arp4 group, which differs slightly from the BioNJ consensus tree in other eukaryotic taxa as well as in the apicomplexans. Relationships were calculated in PAUP*4.01b using the heuristic algorithm and verified by bootstrapping (>100 replicates). Consensus trees were drawn according to the bootstrap 50% majority-rule. Conventional actins were defined as the out-group. Subgroups of Arps and ALPs have been highlighted to define the boundaries between groups. Taxa are defined in Figure 1. Bootstrap values ≥90% are represented by the black nodes ●, values ≥ 75% are represented by the gray nodes , and values ≥50% are denoted by the white nodes ○.

We searched the recently completed genomes of *Toxoplasma gondii, Plasmodium *spp., *Cryptosporidium *spp., and *Theileria *spp. for actin-related proteins using conventional actins and conserved Arp proteins from organisms spanning several phyla including mammals, plants, flies, worms, yeast, and protozoa [see Additional File 1]. BLAST analysis identified over 60 candidate actin-related proteins in total among the apicomplexan genomes examined in this study (Table 1). Reciprocal BLASTP searches using each of these apicomplexan actin-like proteins against the NCBI CDD database revealed that the majority of them contain a conserved actin domain (pfam00022) (Table 1). However, at present individual actin-related protein groups have not been defined by distinct domains or motifs common to members of only one group. Consequently, we sought to establish relationships between the apicomplexan actin-like proteins and conventional Arps using sequence alignment and phylogenetic analyses. Candidate actin-related proteins were aligned with a broader spectrum of Arps from a variety of eukaryotic taxa and bacterial actin-like proteins using CLUSTALX [27]. The relative divergence of actin-like proteins was determined by Neighbor-Joining distance analysis using the phylogenetic analysis program PAUP*4.01b [28]. The resulting bootstrapped phylogram is shown in Fig. 1. Parsimony analysis revealed a similar branching pattern for the major Arp groups, but was less able to resolve deep branching groups (i.e. Arp4 and various apicomplexan specific ALPs), likely due to the divergence of these sequences (Fig. 2). We have focused primarily on the relationships supported by distance analysis, since this methodology is more appropriate for highly divergent sequences.

**Table 1:** Actin-Like Protein (ALP) Family Members in *Toxoplasma gondii *and other Apicomplexans

	***Toxoplasma gondii *(Tg)**				***Plasmodium falciparum *(Pf)**				***Cryptosporidium parvum *(Cp)**				***Theileria parva *(Tp)**			
**ALP Protein**	**Gene ID **^**a**^	% ID to TgACT1 *	***E *-value**	**Pfam score **^**b**^	**Gene ID **^**a**^	% to ID Tg ALP *	***E *-value**	**Pfam score **^**b**^	**Gene ID **^**a**^	% ID to Tg ALP1 *	***E *-value**	**pfam score **^**b**^	**Gene ID **^**a**^	% ID to Tg ALP1 *	***E *-value**	**pfam score **^**b**^
				
**Arp1**	TgTwinScan_4250	53%	1.00 × 10 ^-116^	492	CAD48998	63%	1.00 × 10 ^-145^	464	EAK87959	57%	1.00 × 10 ^-134^	437	-	-	-	-
**ALP1**	AAW23163	39%	4.00 × 10 ^-73^	295	AAN35700	49%	1.00 × 10 ^-104^	257	EAK88581	45%	3.00 × 10 ^-94^	292	EAN34027	39%	3.00 × 10 ^-84^	246
**ALP2a**	TgTwinScan_4277	27%	4.00 × 10 ^-05^	53.4	AAN35636	20%	4.00 × 10 ^-22^	78.4	EAL37900 ^c^	27%	2.00 × 10 ^-13^	85.4	EAN34250	24%	2.00 × 10 ^-24^	67.7
**ALP2b**	-	-	-	-	CAD51417 ^d^			98.1	-	-	-	-	-	-	-	-
**ALP3**	TgTwinScan_2515	23%	2.00 × 10 ^-16^	80.8	CAD51025	36%	1.00 × 10 ^-6^	-	EAK89329	20%	1.00 × 10 ^-3^	73.1	-	-	-	-
**ARP4a**	TgTwinScan_2909	34%	6.00 × 10 ^-19^	114	AAN36831	36%	1.00 × 10 ^-24^	131	EAK89417	38%	1.00 × 10 ^-22^	250	EAN32990	27%	2.00 × 10 ^-38^	160
**ARP4b**	TgTwinScan_6634	27%	1.00 × 10 ^-33^	115	-	-	-	-	-	-	-	-	EAN33438	22%	5.00 × 10 ^-15^	61.9
**ALP5a**	-	-	-	-	CAD51790 ^e^			44.9	-	-	-	-	-	-	-	-
**ALP5b**	-	-	-	-	CAD49164 ^e^			68.4	-	-	-	-	-	-	-	-
**ARP6**	TgTwinScan_6605	20%	4.00 × 10 ^-08^	71.1	CAD50940	40%	3.00 × 10 ^-23^	68	EAL35517 ^c, d^	30%	5.00 × 10 ^-22^	91.9	EAN33600	31%	1.00 × 10 ^-37^	84.6
**ALP7a**	-	-	-	-	-	-	-	-	EAK88375 ^e^			47.6	-	-	-	-
**ALP7b**	-	-	-	-	-	-	-	-	EAK88162 ^e^			110	-	-	-	-
**ALP8**	TgTwinScan_0463 ^f^	25%	3.00 × 10 ^-25^	106	-	-	-	-	-	-	-	-	-	-	-	-
**ALP9a**	TgTwinScan_2686	24%	0.008	46.5	-	-	-	-	-	-	-	-	-	-	-	-
**ALP9b**	TgTwinScan_7210			-	-	-	-	-	-	-	-	-	-	-	-	-

* = BLAST2 pairwise comparison

(-) = no significant match

*E *-value from Pairwise Blast (BLAST 2 sequences)

a = Gene ID obtained from , , , and the NCBI database

b = Pfam score to Pfam domain pfam00022 as determined by BLASTP comparison to the CDD NCBI database

c = *Cryptosporidium hominis*

d = Cp ortholog encoded on contig_AEE01000007 nt# 293712–294650 frame1. Translated using GENESCAN webserver

e = primary family member

f = entire Tg ortholog encoded by TGG_994550 nt# 296106–297827. Translated using GENESCAN webserver

Our analysis reveals that the apicomplexans all encode a single conventional actin (with the exception of *Plasmodium *which has two conventional actins), and the remaining proteins form a total of 10 distinct actin-related protein groups (Fig. 1). Three of these groups were shown to belong to well-characterized Arps including Arp1, Arp4, and Arp6 (Fig. 1). In contrast, we discovered that several other apicomplexan actin-like proteins (ALPs) were unique to this phylum, as they did not group with any of the conventional Arps (i.e. ALP1 and ALP2)(Fig.1); therefore, we have used the designation actin-like protein (ALP) to differentiate the apicomplexan-specific protein groups. A comparison of actin-like proteins within the Apicomplexa is summarized in Table 1. The remaining ALPs were specific to a subset of apicomplexans such as *Toxoplasma *(i.e. ALPs 8, and 9), *Cryptosporidium *(i.e. ALP7) and *Plasmodium *(i.e. ALP5). Several of these groups also contain paralogues, for example ALP5a and ALP5b in *Plasmodium *(Fig.1). While some ALPs appear as deep branches (i.e. TpALP4b, PfALP2b, CpALP7a and CpALP7b, TgALP2a, TgALP3) they were grouped and hence named in part based on BLAST results (Table 1) and phylogenetic analysis of apicomplexan ALPs compared in the absence of other organisms (data not shown). Our findings suggest that some actin-like proteins play roles that are conserved across all eukaryotes while other members of this group have diverged to fulfill specific roles within the Apicomplexa.

The two key features most prominent about the apicomplexan actin-like proteins are their strong conservation of the Arp1 protein (a major component of the dynactin complex) and their remarkable lack of both Arp2 and Arp3 homologues (subunits of the Arp2/3 actin polymerization complex) (Fig. 1). The presence of a highly conserved Arp1 and the absence of Arp2 and Arp3 orthologues have important biochemical implications for these parasites regarding vesicular trafficking and actin regulation, respectively. Arp1 is an essential component of the dynactin complex involved in vesicular trafficking [29,30] while Arp2/3 forms a multi-subunit complex that is the primary means of regulating actin polymerization in eukaryotic cells [31,32]. For these reasons, we conducted a more comprehensive study of the protein components that constitute these complexes.

### The dynactin complex

Dynactin is a microtubule-associated complex that is critical for tethering cellular cargo to the cytoplasmic motor protein dynein [30]. Cytoplasmic dynein consists of heavy, light, intermediate, and intermediate light chains in addition to several regulatory subunits [33]. We searched the *P. falciparum *and *C. parvum *genomes to identify components of this complex using text word searches. Convincing orthologues for all of the subunits were found in both parasites as shown by reciprocal BLASTP and the presence of conserved pfam domains (Table 2). These hits were then used to identify orthologues in other apicomplexan genomes by BLASTP as verified by both significant BLAST *E*-values and the presence of conserved pfam domains (Table 2). Somewhat surprisingly, a complete complex was not readily identified in *Theileria*, with the exception of subunits for heavy and light chains (Table 2).

**Table 2:** Conserved Cytoplasmic Dynein Subunits in Apicomplexans

		***Toxoplasma gondll *(Tg)**				***Plasmodium falciparum *(Pf)**				***Cryptosporidium parvum *(Cp)**				***Thelleria parva *(Tp)**			
**Dynein Subunit**	**Pfam ID**	**Tg Candidate **^**a**^	**Top Match **^**b**^	***E *-value **^**c**^	**Pfam Score **^**d**^	**Pf Candidate **^**a**^	**Top Match **^**b**^	***E *-value **^**c**^	**Pfam Score **^**d**^	**Cp Candidate **^**a**^	**Top Match **^**b**^	***E *-value **^**c**^	**Pfam Score **^**d**^	**Tp Candidate **^**a**^	**Top Match **^**b**^	***E *-value **^**c**^	**Pfam Score **^**d**^
				
**Heavy Chain**	pfam03028	TgTwinScan_0436	Rn heavy chain	0	402	CAD51040	Rn heavy chain	0	297	EAK88498	Dm heavy chain	0	274	CAI73268 ^e, f^	Ce heavy chain	2.00 × 10 ^-76^	76.1
**Light Chain**	pfam01221	TgTwinScan_2634	Mm light chain2	6.00 × 10 ^-24^	148	AAN36221	Rn light chain	1.00 × 10 ^-38^	130	EAL37552 ^g, h^	Dm light chain 1	4.00 × 10 ^-41^	142	EAN33478	Rn light chain	2.00 × 10 ^-27^	111
**Intermediate Chain**	nd	TgTwinScan_1768	Xt intermediate chain	7.00 × 10 ^-80^		AAN35394	Dd intermediate chain	1.00 × 10 ^-82^		EAK88439	Xt intermediate chain	1.00 × 10 ^-48^		-	-	-	-
**Intermediate Light Chain**	pfam05783	TgTwinScan_4175	Hs intermediate light chain	8.00 × 10 ^-07^	63.9	CAD51749	Gg intermediate light chain	3.00 × 10 ^-10^	72	EAK88297	Gg intermediate light chain	0.023	43.5	-	-	-	-
**Dynein Light Chain TcTex1**	pfam03645	TgTwinScan_1459	Hs TcTex1	2.00 × 10 ^-12^	81	CAD51956	Hs TcTex1	1.00 × 10^-12^	70.3	EAK87898	Mm TcTex1	9.00 × 10 ^-17^	83.4	-	-	-	-
**Roadblock**	pfam03259	TgTwinScan_6940	Ci roadblock	6.00 × 10 ^-34^	81.8	AAN35393	Ci roadbloack	1.00 × 10 ^-24^	85.3	EAK88245	Ci roadblock	0.24	-	-	-	-	-

nd = not defined

(-) = no significant match

a = gene candidates identified by text and BLASTP searches of , , , and the NCBI nr database

b = identifying protein found in reciprocal BLASTP search of the NCBI nr database using apicomplexan candidates as query

c = *E *-value of reciprocal BLASTP search

d = Pfam score as determined by BLASTP comparison to the CDD NCBI database

e = *Theileria annulata*

f = Tp ortholog EAN34073

g = *Cryptosporidium hominis*

h = Cp ortholog encoded on contig_AAEE01000005 nt#75605–75871frame1. Translated using GENESCAN webserver

Ce = *Caenorhabditis elegans, Ci = Ciona intestinalis, Dd = Dictyostelium discoideum, Dm = Drosophila melanogaster, Hs = Homo sapien, Gg = Gallus gallus*, Mm = *Mus musculus, Rn = Rattus norvegicus*, Xt = *Xenopus tropicalis*

Based on the presence of a conserved dynein complex in a majority of apicomplexans, we thought it reasonable to search for evidence of a dynactin complex. The dynactin complex consists of several protein subunits that are grouped into two domains: the Arp1 rod and a flexible arm region. The protein subunits of the Arp1 rod are more highly conserved between eukaryotes than the remaining dynactin subunits [30]; therefore, we focused our efforts on defining homologues to these proteins in the apicomplexans. The subunits comprising the Arp1 rod include Arp1, Arp11, capping protein (CapZ), p62, p25, p27, and actin (see [30] for a complete review of the dynactin complex).

We used database searches to identify the dynactin subunits within apicomplexans. Sequences from mammals, flies, worms, and protozoa were compared against the NBCI nr database and the respective genomic databases of *Toxoplasma, Plasmodium*, and *Cryptosporidium *(Table 3) [see Additional file 1]. Arp1 was readily identified in *Plasmodium, Cryptosporidium*, and *Toxoplasma*, although it is apparently absent in *Theileria *(Table 1). Highly conserved orthologues of the p25, p27, and p62 subunits were found in *Toxoplasma, Plasmodium*, and *Cryptosporidium *as shown by both significant BLASTP *E*-values and the presence of conserved pfam domains (Table 3).

**Table 3:** Conserved Dynactin Subunits in Apicomplexans

		***Toxoplasma gondii *(Tg)**				***plasmodium falciparum *(Pf)**				***Crytosporidium parvam *(Cp)**			
**Dynactin Subunit**	**Pfam ID**	**Tg Candidate **^**a**^	**Top Match **^**b**^	***E *-value **^**c**^	**Pfam score **^**d**^	**Pf Candidate **^**a**^	**Top Match **^**b**^	***E *-value **^**c**^	**Pfam score **^**d**^	**Cp Candidate **^**a**^	**Top Match **^**b**^	***E *-value **^**c**^	**Pfam score **^**d**^
			
Arp1 *	pfam00022	TgArp1 TgTwinScan_4250	GgArp1	1.00 × 10 ^-147^	492	PfArp1 CAD48998	Gg Arp1	1.00 × 10^-141^	464	CpArp1 EAK87959	GgArp1	1.00 × 10 ^-131^	437
Arp10/11 *	pfam00022	TgALP3 TgTwinScan_2515	Gg Arp10	8.00 × 10 ^-28^	80.8	PfALP3 CAD51025	Dr Arp10	8.00 × 10 ^-05^	-	CpALP3 EAK89329	GgArp10	9.00 × 10 ^-21^	73.1
**p62 **^**e**^	pfam05502	TgTwinScan_5099	Hs p62	3.00 × 10 -^09^	62.8	AAN37118	Hs p62	5.00 × 10 ^-05^	56.7	EAK88826	Hs p62	9.00 × 10 ^-21^	101
**p25 **^**f**^	nd	TgTwinScan_4906	Gg p25	4.00 × 10 ^-29^		CAD50982	Dd p25	2.00 × 10 ^-21^		EAK87596	Dd p25	9.00 × 10 ^-18^	
**p27 **^**g**^	nd	TgTwinScan_1451	Sp p27	8.00 × 10 ^-05^		CAD51191	Bt p27	0.009		EAK90307	Am p27	9.00 × 10 ^-08^	
**CapZ **α	pfam01267	AAU93918	At CapZ α	1.3	-	CAD51646	Dd CapZ α	4.00 × 10 ^-17^	102	-	-	-	
**CapZ **β	pfam01115	AAU93916	Dm CapZ β	2.00 × 10 ^-45^	197	CAD51540	Dm CapZ β	4.00 × 10 ^-29^	140	EAK88546	Dd CapZ β	2.00 × 10 ^-07^	53.9
**Dynamitin/p5O **^**h**^	pfam04912	TgTwinScan_4110	Dr p5O	4.00 × 10 ^-10^	62	CAD52583	XI p5O	1.00 × 10 ^-04^	52.8	-	-	-	-

* = see phylogenetic analysis for definition of apicomplexan candidates

nd = not defined

(-) = no significant match

a = protein ID of candidate apicomplexan protein

b = identifying protein found in reciprocal search of the NCBI database using the apicomplexan candidate as query

c = *E *-value of the top match identifying protein in comparison to the apicomplexan candidate

d = Pfam score as determined by BLASTP comparison to the CDD NCBI database

e = protein sequences used to identify apicomplexan candidates : Hs AAH26323, Dm AAF59211, Ce AAC24257, Dd XP_641285

f = protein sequences used to identify apicomplexan candidates: Hs Q9BTE1, Dm AAF34709, Sp XP_782293, Dd EAL68462

g = protein sequences used to identify apicomplexan candidates : Hs AAH13175, Dm NP_609949, Ce NP_491116

h = protein sequences used to identify apicomplexan candidates : Hs AAC50423, Dm AAF59034, Ce NP_498286, Dd XP_638093

Am = *Apis melliferous*, At = *Arabidopsis thaliana*, Bt = *Bos taurus*, Ce = *Caenorhabditis elegans*, Dd = *Dictyostelium discoideum*, Dm = *Drosophila melanogaster*, Dr = *Danio rerio*, Hs = *Homo sapiens*, Gg = *Gallus gallus*, Sp = *Strongylocentrotus purpuratus*, XI = *Xenopus laevis*

The Arp1 rod contains a short filament of Arp1 subunits [34] that is capped at both ends. The (+) or barbed end is terminated by capping protein [35] and the (-) or pointed end by the actin-related protein Arp11 [36]. *Toxoplasma *and *Plasmodium *both contain β subunits of capping protein, and the α subunit in *Plasmodium *showed a significant BLASTP *E*-value and conserved pfam motif (Table 3). The α subunit reported for *Toxoplasma *is highly divergent (NCBI AAU93918) and does not have significant matches, although BLASTP searches turn up a number of α subunit orthologues (Table 3). Additionally only the β subunit was identified in *Cryptosporidium *(Table 3). Capping protein always exists as an α/β dimer [37] and it is possible the α subunit is divergent in *Toxoplasma *and *Cryptosporidium *and hence difficult to recognize at present. Our phylogenetic analysis of the Arps did not show strong affinities between any of the ALP proteins and the Arp11 group (Fig. 1). However, we have included TgALP3, PfALP3, and CpALP3 as possible Arp11 orthologues based on their sequence similarity to the Arp11 proteins in BLASTP searches (Table 3).

Dynamitin is a component of the flexible arm region of the dynactin complex [30]. We identified proteins with recognizable dynamitin domains in *Toxoplasma *and *Plasmodium*, but not *Cryptosporidium *(Table 3). The remaining subunits of the dynactin complex were not detected by BLAST or protein domain searches in these organisms. However since these other subunits are less well conserved, failure to detect them by BLAST is not surprising.

The identification of apicomplexan orthologues to all the subunits of the Arp1 rod, and the presence of dynamitin in *Toxoplasma *and *Plasmodium*, provides strongly supportive evidence that a functional complex exists in these parasites. *Theileria *appears to be an exception to this pattern as neither Arp1 or the other subunits were recognized. The conserved complex in parasites likely carries out duties analogous to the dynactin in other eukaryotes. One possible role for this complex would be the directed delivery of secretory protein vesicles as has been described in other systems [29]. Secretory protein trafficking occurs via an ER-Golgi mediated pathway [38] and dynactin could provide the transportation by which cargo vesicles reach their specialized secretory organelles at the apical pole. Apical secretion is an important component of cellular invasion and maintenance of this polarization is thus vital to the survival of the parasite.

### The Arp2/3 actin polymerization complex

The Arp2/3 complex consists of 7 subunits that regulate actin polymerization at the leading edge in motile cells [23], as well as providing a propulsive force to move endosomes throughout the cytoplasm [39,40]. Arp2/3 is a major nucleator of actin polymerization in most eukaryotic cells; however, our phylogenetic analyses of the apicomplexan actin-related proteins did not show homologues to either Arp2 or Arp3 (Fig. 1). Notably, Arp2 and Arp3 homologues have been previously annotated in both the *Plasmodium *and *Cryptosporidium *genome databases (PfArp3: CAD51790, PfArp2: CAD49164, CpArp3: EAK88375, and CpArp2, EAK88162). These proteins correspond to our annotations PfALP5a, PfALP5b, CpALP7a, and CpALP7b, respectively. Phylogenetic comparisons do not support these previously proposed annotations, but rather indicate that these actin-like proteins are part of other ALP groups (Fig. 1).

A recent analysis of the actin family from model organisms was utilized to derive predictive models for grouping Arp groups in a variety of taxa [41]. Importantly, this analysis also found Arp1, Arp4, and Arp6 homologues among the Apicomplexa (*Plasmodium *and *Cryptosporidium *genomes) but failed to identify orthologues of Arp2, Arp3 or other Arp groups [41]. Collectively, these findings indicate apicomplexans do not encode a conserved Arp2/3 complex.

We also searched for the other 5 subunits of the Arp2/3 complex that are known as actin-related protein complex 1 (ARPC1)/p41, ARPC2/p34, ARPC3/p21, ARPC4/p20, and ARPC5/p16. A separately recognized domain is only described for ARPC4/p20 (pfam05856), perhaps reflecting the divergence of the remaining subunits across the many taxa where they are readily identified by BLAST. We conducted genome-wide BLAST searches of apicomplexans as described above using ARPC proteins from mammals, flies, yeast, plants, and protozoa (see Table 4) [see Additional file 1]. No proteins with similarity to subunits ARPC2, 3, and 5 were found in any of the four apicomplexan genomes.

**Table 4:** Conserved Arp2/3 Complex Subunits in Apicomplexans

	***Toxoplasma gondii *(Tg)**			***Plasmodium falciparum *(Pf)**			***Cryptosporidium parvum *(Cp)**			***Theileria parva *(Tp)**		
**Arp2/3 complex subunit**	**Tg candidate **^**a**^	**Top Match **^**b**^	***E *-value **^**c**^	**Pf candidate **^**a**^	**Top Match **^**b**^	***E *-value **^**c**^	**Cp candidate **^**a**^	**Top Match **^**b**^	***E *-value **^**c**^	**Tp candidate **^**a**^	**Top Match **^**b**^	***E *-value **^**c**^
				
**Arp2**	-	-	-	-	-	-	-	-	-	-	-	-
**Arp3**	-	-	-	-	-	-	-	-	-	-	-	-
**ARPC1/ p41 **^**d**^	-	-	-	AAN35779	SpARPC1	0.002	EAK89688	MmARPC1	1.00 × 10 ^-11^	-	-	-
**ARPC2/p34 **^**e**^	-	-	-	-	-	-	-	-	-	-	-	-
**ARPC3/ p21 **^**f**^	-	-	-	-	-	-	-	-	-	-	-	-
**ARPC4/ p20 **^**g**^	-	-	-	-	-	-	EAK89016	OsARPC4	4.00 × 10 ^-11^	-	-	-
**ARPC5/ p16 **^**h**^	-	-	-	-	-	-	-	-	-	-	-	-

(-) = no significant match

a = protein ID of candidate apicomplexan protein

b = identifying protein found in reciprocal BLASTP search of the NCBI database using the apicomplexan candidate as query

c = *E *-value of the top match identifying protein in comparison to the apicomplexan candidate

d = protein sequences used to identify apicomplexan candidates : Hs Q92747, Dm CAB38634, Sc P38328, At AAO42862, Dd AAC99777, Tc EAN83660

e = protein sequences used to identify apicomplexan candidates : Hs NP_690601, Dm Q9VIM5, Sc NP_014433, At AAM60850, Dd AAC99778, Tc EAN93128

f = protein sequences used to identify apicomplexan candidates : Hs AAH67747, Dm NP_013474, Sc NP_013474, At AAM61177, Dd AAC99779, Tc EAN89964

g = proteins used to obtain apicomplexan candidates : Hs AAB64192, Dm AAF52346, Sc NP_012912, Dd AAC99780, Tc XP_810627

h = protein sequences used to identify apicomplexan candidates : Hs NP_005708, Dm NP_608693, Sc P40518, Dd AAC99781, Tc EAN80710

At = *Arabidopsis thaliana*, Dd = *Dictyostelium discoideum*, Dm = *Drosophila melanogaster*, Hs = *Homo sapiens*, Mm = *Mus musculus*, Os = *Oryza sativa*, Sc = *Saccharomyces cerevisiae*, Sp = *Strongylocentrotus purpuratus*, Tc = *Trypanosoma cruzi*

Potential orthologues to the ARPC1/p41 were found in *Plasmodium *and *Cryptosporidium *(Table 3): both of these proteins contain WD40 repeats, which are a distinguishing feature of the ARPC1/p41 proteins in other eukaryotes [42]. This analysis was supported by BLAST and also by protein domain searches using Prosite, which identified WD40 repeat domains in both proteins (Pfam PF00400, SMART domain SM00320). WD40 repeats mediate protein-protein interactions and are involved in regulating numerous biological functions in addition to their role in actin nucleation [43,44]. Since ARPC1/p41 is not necessary for the overall cohesiveness of the Arp2/3 subunits [45], we can hypothesize this protein may serve an alternative function outside of the Arp2/3 complex in *Plasmodium *and *Cryptosporidium.*

Surprisingly, *Cryptosporidium *encodes a conserved ARPC4/p20 subunit as shown by BLAST analysis and by Prosite domain similarity (pfam PF05856) (Table 4). In other eukaryotes, ARPC4 forms a stable heterodimeric complex with ARPC2/p34 that comprises the structural core of the Arp2/3 complex [45]. In the absence of ARPC4/p20, Arp2/3 complexes are not formed [45], underscoring its importance to the protein scaffold. It is therefore unusual that *Cryptosporidium *would retain a close orthologue to one subunit and completely lack the other (Table 4). Additionally, the ARPC2/ARPC4 heterodimer binds actin filaments and is thought to be necessary for branching of daughter filaments from existing mother filaments [45]. Actin in *Toxoplasma *does not appear to be branched [46], thus it is unclear why *Cryptosporidium *maintains an ARPC4 homologue (Table 4).

The presence of remnant ARPC1/p41 homologues in *Plasmodium *and *Cryptosporidium*, and ARPC4/p20 in *Cryptosporidium *indicates that the complex may have been functional at one time in these parasites; however, they either have since lost the complex completely or the subunits have diverged to the extent that they are no longer recognizable. Support for this hypothesis comes from other alveolates, such as the closely-related but deeper branching ciliate lineages [47]. The ciliate *Tetrahymena thermophilia *encodes a canonical Arp2/3 complex with easily recognizable Arp2 (AAN73249), Arp3 (AAN73250), ARPC2 (4.m00362), ARPC3 (43.m00326) and ARPC4 (152.m00065) subunits. Loss of a functional complex in the apicomplexans may have resulted from their highly specialized, intracellular lifestyles. Deciphering how apicomplexans control actinfilament turnover is thus an intriguing and unanswered question. We postulate that evolution of alternative proteins, such as the ALP1 proteins (Fig. 1), could enable parasites to regulate actin polymerization in a more streamlined mechanism, yet maintain the overall function of the complex.

The ALP1 group of apicomplexan-specific proteins is phylogenetically similar to both the Arp2 and Arp3. ALP1 in *Toxoplasma *is the second closest paralogue to conventional actin with which it shares 37% identity and 57% similarity (Table 1). Moreover, TgALP1 is 49% identical to PfALP1 and 45% identical to CpALP1, indicating the ALP1 proteins are highly conserved within this phylum (Table 1). These phylogenetic properties, in conjunction with the lack of any obvious Arp2 or Arp3 homologues, lead us to hypothesize that the ALP1 proteins may play a corresponding or complementary role to these two proteins in the apicomplexans.

### Arps and chromatin remodeling

In addition to their cytoskeletal roles, actin and Arps function in the nucleus as components of chromatin-modifying and chromatin-remodeling protein complexes [48]. These Arps include Arp4, Arp5, Arp6 and Arp8 [26,49-51]. Arps 7 and 9 are yeast-specific and do not have homologues in other eukaryotes [48]. Our studies show the apicomplexans encode conventional Arp4 and Arp6 orthologues (Fig. 1).

Chromatin-modifying and -remodeling machinery are involved in DNA replication, DNA repair mechanisms and transcriptional regulation [52]. Arp4 is present in several complexes including the NuA4 histone acetyltransferase and several members of the ATP-dependent SWI2-SNF2 family of chromatin-remodeling complexes [51,53]. In yeast, Arp6 is also a member of SWR1, a subgroup of the SWI2/SNF2 chromatin-remodeling complexes [50]. Other roles for Arp6 include transcriptional deactivation via heterochromatin-remodeling in *Drosophila *and vertebrates [24].

Changes in gene expression are important means of regulating function and such changes have been shown to play a role in parasite stage-differentiation [54-56]. The *Plasmodium *and *Cryptosporidium *genomes appear to lack many common transcription factors leading to the hypothesis that these parasites rely heavily on chromatin-remodeling for transcriptional control [57,58]. This is supported by the fact that apicomplexans appear to contain several components of the SWI2/SNF2 ATPase chromatin-remodeling machinery [57,58]. Recently, Saksouk *et. al*. showed the first direct correlation of histone acetylation and methylation to stage-specific gene expression in *Toxoplasma *[59], supporting the importance of chromatin modification and remodeling in these parasites. The presence of conserved Arp4 and Arp6 orthologues suggests that actin-related proteins participate in chromatin remodeling in apicomplexans similar to other eukaryotes.

## Conclusion

Comprehensive analysis of the genome content of these parasites combined with phylogenetic groupings has allowed us to propose potential functions for many of these Arp/ALP groups. Our findings indicate that apicomplexans encode a variety of actin-like proteins (ALPs) that likely participate in actin-based motility, vesicle transport, and transcriptional regulation through chromatin remodeling. Delineating their respective functions will ultimately enrich our understanding of these parasites, and also the evolution of the actin family as a whole.

## Methods

### Assembly of actin-like proteins from apicomplexans and other alveolates

Comprehensive BLAST searches were performed against the *T. gondii *genome database (ToxoDB Release v3.0) using 27 protein sequences from Arp1 through Arp4 that represented major taxa including mammals, plants, flies, worms, yeast, and protozoa. Actin-like proteins were identified in *Plasmodium *spp. (PlasmoDB Release v4.3) and *C. parvum *(NCBI nr database and CryptoDB Release v3.0) by combination of tBLASTn and BLASTP searches using the above conserved Arps or Toxoplasma candidate actin-like sequences. BLAST searches were restricted to only high quality "hits" (e-value of ≤ .0001). In the case where only nucleotide data was available, the matching nucleotide sequence was translated using the GENESCAN webserver [60] using *Arabidopsis thaliana *to predict exon-intron structures. In these cases, the resulting amino acid sequence predictions were used in all subsequent analyses. Once identified, candidate sequences were entered into a "reverse" BLAST search of the NCBI database [61] to determine if there was a reciprocal best match to the protein used to identify it.

Protein candidates from *Tetrahymena thermophila *were obtained via BLASTP searches of the NCBI nr database comparing Arps from model organisms and by searching the *Tetrahymena *genome database [62]. Searches of the *Tetrahymena *genome database were done using tBLASTn and restricted to TIGR predicted proteins.

Preliminary sequence data was obtained from The Institute for Genomic Research website [63], ToxoDB [64], PlasmoDB [65], CryptoDB [66].

A complete list of all taxa and accession/contig numbers used in these studies is provided [see Additional file 1].

### CLUSTALX alignments

The above candidate actin-like protein sequences were compared with a larger repertoire of Arp proteins from a variety of eukaryotes [67] and bacterial actin-like proteins retrieved from NCBI. All sequences were entered into the alignment program CLUSTALX [27] using pairwise parameters set as: gap opening penalty = 15.0, gap extension penalty = 0.10; and multiple alignment parameters set as: gap opening penalty = 15.0, gap extension penalty = 0.30, delay divergent sequences (%) = 25. All other parameters were set to the default settings. Clustal alignments used in this analysis are posted at [68].

### Phylogenetic analysis

CLUSTAL alignments were entered into the phylogenetic analysis program PAUP4.0b10 for Macintosh [28]. Only regions of the alignments with conservation across all taxa were included in the analyses. The optimality criterion was set to distance (mean character difference, minimal evolution, negative branches = 0) and 1000 bootstrap replicates were performed using the BioNeighbor-Joining (BioNJ) algorithm. Alternatively, a full heuristic algorithm was used for parsimony analysis, supported by bootstrapping for > 100 replicates. Consensus trees were drawn according to the Bootstrap 50% majority-rule and conventional actins were defined as the out-group.

### Dynactin and Arp2/3 complex subunits

Highly conserved subunits of both the dynactin and Arp2/3 complexes were retrieved from NCBI nr for model organisms [see Additional file 1]. These proteins were used in BLASTP searches of the *Toxoplasma *[64], *Plasmodium *[65], *Cryptosporidium *[61,66], *Theileria *[61], and *Tetrahymena *[61,62] databases for candidate orthologues, as described above. Candidate proteins were used in a "reverse" BLAST of the NCBI database [61] to determine their relatedness to the proteins used to identify them.

## List of abbreviations

ALP, Actin-like protein; Arp, actin-related protein; ARPC, actin related protein complex; capZ, capping protein; CDD conserved domain database, NCBI, National Center for Biotechnology Information, pfam, protein family database.

## Authors' contributions

JLG and LDS devised the overall strategy for these studies. JLG performed all database analyses, sequence alignments, and phylogenetic comparisons. JLG authored the text of this manuscript and LDS provided comments and revisions to the final version of this text.

## Supplementary Material

Additional File 1 Taxa. Listing of the taxa and accession numbers for protein alignments and BLAST analyses used in the present study. Click here for file
