# Supplementary material for: Comparative genome analysis reveals a conserved family of actin-like proteins in apicomplexan parasites
Source: BMC Genomics. 2005 Dec 12;6:179. doi: 10.1186/1471-2164-6-179 (PMC1334187; doi:10.1186/1471-2164-6-179)
Supplement: Additional File 1 — Taxa. Listing of the taxa and accession numbers for protein alignments and BLAST analyses used in the present study. [file 1471-2164-6-179-S1.pdf]

Additional Files

Taxa and protein accession numbers for actin and related proteins included in phylogenetic analysis are listed below:

Actin: Tg P53476, Pf AAN36527, Pf AAN36736, Py EAA21675, Py EAA20889, Cp AAM28417, Ch EAL35958, Tp EAN33188, Ta CAI74081, Tp EAN32342, Ta CAI74918, Pm AAR11391, Dm P45891, Sc NP_116614, At NP_181740, Dd P02577

Arp1: Pf CAD48998, Tg TwinScan_4250, Py EAA15967, Cp EAK87959, Ch EAL36700, Sc NP_011997, Ce NP_497108, Dm P45889, Hs NP_005726, Hs NP_005727

Arp2: At NP_189336, Sc P32381, Dd AAC99776, Tt AAN73249, Ce NP_505657, Dm S47987, Hs AAH14546

Arp3: At NP_172777, Tt AAN73250, Sc NP_012599, Dd P42528, Ce NP_491066, Dm NP_523968, Hs AAH14546

Arp4: Pf AAN3683, Py EAA21218, Cp EAK89417, Ch XP_667364, Tp EAN32990, Ta CAI74275, Tg TwinScan_6634, Tp EAN33438, Ta CAI73847, Tg TwinScan_2909, Sc NP_012454, At NP_564051, Ce NP_500801, Dm NP_611209, Hs BAA74577

Arp5: At NP_566422, Hs CAD37358, Dm NP_650684, Sc P53946

Arp6: Tg TwinScan_6605, Pf CAD50940, Py EAA21903, Cp contig AEE01000007_293712-294650, Ch EAL35517, Tp EAN33600, Ta CAI73702, Sc NP_013186, At NP_566861, Ce NP_495681, Dm NP_511165, Hs Q9GZN1

Arp7: At NP_567105, Sc Q12406

Arp8: At NP_568836, Dm NP_573251, Hs NP_075050

Arp9: Sc NP_013747

Arp10: Sc NP_010391, Dm NP_608338

Arp11: Hs Q9NZ32

ALP1: Tg AAW23163, Pf AAN35700, Py EAA21110, Cp EAK88581, Ch EAL37105, Tp EAN34027, Ta CAI73309

ALP2a: Tg TwinScan_4277, Pf CAD51417, Py EAA19047, Ch EAL37900, Tp

EAN34250, Ta CAI73086

ALP2b: Pf AAN35636, Py EAA19205

ALP3: Pf CAD51025, Py EAA20604, Cp EAK89329, Ch EAL36230, Tg TwinScan_2515

ALP5a : Pf CAD51790, Py EAA18953

ALP5b: Pf CAD49164, Py EAA15905

ALP7a: Cp EAK88375, Ch contig AAEL01000022_22753-23685

ALP7b: Cp EAK88162, Ch contig AAEL01000517_3524-4378

ALP8: Tg 83.m01292

ALP9a: Tg TwinScan_2686

ALP9b: TgTwinScan_7210

Bacterial actin-like proteins: Bs NP_390681, Bs P39751

Miscellaneous actin-like proteins: Tt AAN73251

Tetrahymena TIGR gene predictions: Tt200.m00048, Tt39.m00235, Tt75.m00216, Tt181.m00072, Tt31.m00302, Tt192.m00074, Tt10.m00319, Tt30.m00196, Tt27.m00277

Taxa and protein accession numbers for dynactin and Arp2/3 complex subunits included in database analyses are listed below:

Dynactin Complex:

Dynamitin/p50: Hs AAC50423, Dm AAF59034, Ce NP_498286, Dd XP_638093, TgTwinScan_4110, Pf CAD52583

p62: Hs AAH26323, Dm AAF59211, Ce AAC24257, Dd XP_641285, TgTwinScan_5099, Pf AAN37118, Cp EAK88826

p25: Hs Q9BTE1, Dm AAF34709, 2Sp XP_782293, Dd EAL68462, TgTwinScan_4906, Pf CAD50982, Cp EAK87596

p27: Hs AAH13175, Dm NP_609949, Ce NP_491116, TgTwinScan_1451, Pf CAD51191, Cp EAK90307

Arp2/3 Complex:

ARPC1/p41: Hs Q92747, Dm CAB38634, Sc P38328, At AAO42862, Dd

AAC99777, Tc EAN83660, Pf AAN35779, Cp EAK89688

ARPC2/p34: Hs NP_690601, Dm Q9VIM5, Sc NP_014433, At AAM60850, Dd AAC99778, Tc EAN93128

ARPC3/p21: Hs AAH67747, Dm NP_013474, Sc NP_013474, At AAM61177, Dd AAC99779, Tc EAN89964

ARPC4/p20: Hs AAB64192, Dm AAF52346, Sc NP_012912, Dd AAC99780, Tc XP_810627, Cp EAK89016

ARPC5/p16: Hs NP_005708, Dm NP_608693, Sc P40518, Dd AAC99781, Tc EAN80710

Abbreviations of taxa:

At=*Arabidopsis thaliana*, Bs =*Bacillus subtilis*, Ce=*Caenorhabditis elegans*, Ch=*Cryptosporridium homis,* Cp=*Cryptosporidium parvum*, Dd=*Dictyostelium discodium*, Dm=*Drosophila melanogaster*, Hs=*Homo sapiens*, Lm=*Leishmania major*, Pf=*Plasmodium falciparum,* Py=*Plasmodium yoelii* , Sc=*Saccharomyces cerevisiae*, Ta=*Theileria annulata,* Tb=*Trypanosoma brucei*, Tc=*Trypanosoma cruzi,* Tg=*Toxoplasma gondii*, Tp=*Theileria parva,* Tt=*Tetrahymena thermophila*
